# The Role of Chemokine (C‐C Motif) Ligand 7 (CCL7) in Hepatocellular Carcinoma: Expression, Function, and Mechanisms

**DOI:** 10.1002/cam4.70701

**Published:** 2025-03-10

**Authors:** Yangkun Luo, Fei Wan, Zhao Zhang, Zujun Qin, Yongjun Ren

**Affiliations:** ^1^ Department of Radiology Affiliated Hospital of North Sichuan Medical College Nanchong Sichuan China; ^2^ Department of Radiology Sichuan Province Orthopedic Hospital Chengdu Sichuan China; ^3^ Department of Hepatobiliary Surgery Affiliated Hospital of North Sichuan Medical College Nanchong Sichuan China; ^4^ Department of Pathology Affiliated Hospital of North Sichuan Medical College Nanchong Sichuan China

**Keywords:** cancer progression, CCL7, hepatocellular carcinoma, PI3K/AKT pathway

## Abstract

**Aim:**

AHepatocellular carcinoma (HCC) is a malignant neoplasm characterized by a poor prognosis, with its incidence rising globally. Chemokine (C‐C motif) ligand 7 (CCL7), a chemokine protein, has been implicated in the progression of various cancers. Nonetheless, the specific role of CCL7 in HCC has yet to be elucidated. This study seeks to examine the expression and functional role of CCL7 in the context of HCC.

**Materials & Methods:**

Western blot and immunohistochemistry were used to detect the expression of CCL7 in HCC tissues and cell lines. Cell Counting Kit 8 (CCK8) assay, clonogenic assay, and transwell assay were performed to examine the effects of CCL7 on SNU‐878 cells. Immunofluorescence was used to analyze the expression of proteins associated with epithelial interstitial transformation (EMT). Western blot was used to detect the activation of the PI3K/AKT pathway. In vivo tumorigenesis experiments were performed to assess the role of CCL7 in HCC tumorigenesis.

**Results:**

The results showed that the expression of CCL7 was up‐regulated in HCC tissues, and exogenous CCL7 promoted the proliferation, migration, invasion, and EMT of SNU‐878 cells and VEGF secretion by SNU‐878 cells. Furthermore, CCL7 stimulated the activation of the PI3K/AKT pathway. Further analysis revealed that CCL7 targeted CCR1 and CCR2 to enhance the growth, and metastasis of SNU‐878 cells and VEGF secretion by SNU‐878 cells. CCR1/CCR2 silencing prevented CCL7 from activating the PI3K/AKT signaling pathway in SNU‐878 cells. Moreover, CCL7 facilitated HCC tumorigenesis and VEGF expression in vivo.

**Conclusion:**

Our findings indicate that CCL7 plays a promoting role in HCC growth and tumorigenesis, potentially via targeting CCR1 and CCR2 and activating the PI3K/AKT pathway.

## 1 Introduction

Primary liver cancer, recognized as a common malignant neoplasm worldwide, is experiencing a rising incidence and mortality rate. Hepatocellular carcinoma (HCC) accounts for approximately 85%–90% of all primary liver cancer cases [1]. The disease is characterized by a subtle onset, a high degree of malignancy, and a significant tendency for metastasis and recurrence, all of which contribute to its poor prognosis [2, 3]. The disease is characterized by a subtle onset, a high degree of malignancy, and a significant tendency for metastasis and recurrence, all of which contribute to its poor prognosis [4].

Chemokine (C‐C motif) ligand 7 (CCL7), known as monocyte chemotactic protein‐3 (MCP‐3), is a chemokine widely expressed in fibroblasts, monocytes, and tumor cells [5]. It has multiple functions, such as monocyte recruitment, induction of calcium ion inflow, and promotion of tumor metastasis through interaction with specific receptors CCR1, CCR2, and CCR3 [6, 7]. Based on the variation in cysteine residues, chemokine ligands can be classified into three types: CC, CXC, and XC. Among them, CCL7 belongs to the CC chemokine family and plays a crucial role in physiological and pathological processes [8]. Under normal physiological conditions, it facilitates immune cell migration and localization [9, 10]. However, during pathological processes such as inflammation and tumorigenesis, there is an upregulation in both the expression level and activity of CCL7, which further promotes inflammatory response and tumor development [11]. A previous study showed that CCL7 regulated invadopodia maturation and matrix metalloproteinase (MMP)‐9 mediated collagen degradation in liver‐metastatic carcinoma cells [12]. At present, the precise role and underlying mechanism of CCL7 in HCC remain unclear.

The binding of chemokine to its receptor triggers the activation of intracellular signaling pathways, including the extracellular signal‐regulated kinase (ERK) [13], c‐Jun NH2‐terminal protein kinase (JNK) [14], and phosphatidylinositol‐4,5‐bisphosphate 3‐kinase (PI3K)/protein kinase B (AKT) signaling pathways [15]. The PI3K/AKT signaling pathway is an important intracellular signaling pathway, which plays an important role in the regulation of cell proliferation, migration, and invasion [16, 17]. In liver cancer, abnormal activation of the PI3K/AKT signaling pathway was closely related to tumor occurrence, development, metastasis, and treatment tolerance. Accumulation of phosphorylated PI3K and phosphorylated AKT attenuated the antitumor effect of sorafenib in HCC [18]. Activation of the PI3K/AKT signaling pathway promoted the proliferation and invasion of SMMC‐7721 and BEL‐7402 cells [19]. Up to now, whether the PI3K/AKT signaling pathway mediates the function of CCL7 in HCC progression has not been investigated.

In this study, HCC cell lines and mouse models were used to investigate the function and mechanism of CCL7 in regulating HCC proliferation, migration, and invasion. It was assumed that downstream receptors in HCC cells were activated by CCL7, further enhancing PI3K/AKT signaling activity and aggravating HCC progression.

## 2 Methods and Materials

### 2.1 Clinical Specimens

The study protocols were approved by the Medical Ethics Committee of the Affiliated Hospital of North Sichuan Medical College (File Number: 2023ER330‐1). We collected HCC tissues (*n* = 50), adjacent non‐tumor tissue samples (*n* = 49) and portal vein tumor thrombus (*n* = 10) from patients who underwent surgery at the Affiliated Hospital of North Sichuan Medical College after obtaining written informed consent from them. All tumor specimens were from the surgical resection. None of the patients had received chemotherapy or radiotherapy before tumor excision. All specimens were collected after obtaining informed consent from all patients. All methods were performed following the relevant guidelines and regulations. The pathological or normal state of the samples was confirmed by histopathological and clinical examination.

### 2.2 Animals

Twelve female nude mice (SPF grade, 4 weeks) were purchased from Dashuo Animal Experiment Co. Ltd. (Chengdu, Sichuan). The feeding environment was 24°C ± 1°C, relative humidity 55% ± 5%, and light/darkness for 12 h circulation. The mice were allowed to eat and drink freely. All animal experimental protocols were approved by the Medical Ethics Committee of the Affiliated Hospital of North Sichuan Medical College (File Number: 2023ER330‐1). All methods were carried out according to relevant guidelines and regulations. All methods are reported by ARRIVE guidelines (https://arriveguidelines.org/) for the reporting of animal experiments.

### 2.3 In Vivo Tumorigenesis Test

The BALB/c nude mice were randomly divided into 2 groups (*n* = 6), namely the control group and the CCL7 group. The SNU‐878 cells (5 × 10^6^ cells) were mixed with Matrigel and subcutaneously injected into the armpits of the mice. Then, the nude mice were fed normally, and observed at the inoculation site for leaks. After 7 days, the animals with obvious subcutaneous tumors were grouped and treated. The CCL7 group nude mice were given intratumoral injections of 0.5 μg/time only of human recombinant CCL7 protein three times a week for 3 weeks. The control group was injected with the same amount of normal saline. Tumor volume was measured every 3 days (long diameter × short diameter × short diameter × 1/2). 39 days after the successful establishment of the model, the mice were anesthetized with 5% isoflurane and tumors were weighed.

### 2.4 Immunohistochemistry (IHC) Staining

After paraffin sections were dewaxed to water, the sections were immersed in citrate buffer for antigen repair. Then they were incubated in a wet box, incubated at room temperature for 10 min with drops blocking endogenous peroxidase, and washed with PBS 3 times. Then bovine serum was added and sealed at room temperature for 20 min. PBS was added to the slices with a certain proportion of primary antibody (CCL7, Ki‐67, and VEGF), and the slices were placed flat in a wet box at 4°C and incubated overnight. After washing with PBS 3 times, the secondary antibody was added, and the mixture was incubated at 37°C for 30 min, and then washed with PBS. Fresh DAB color developing solution was added to the tissue, and it was incubated at room temperature for 5 min, and finally dyed with hematoxylin for 3 min, dehydrated, and sealed the tablet. The slices were captured using a microcamera system (BA400Digital; Mike Audi Industrial Group Ltd., Xiamen, China).

### 2.5 Cell Culture

Human hepatocellular carcinoma cell SNU‐878 was purchased from iCell Bioscience Inc. (#iCell‐h458, Shanghai, China). The cells were maintained in 1640 culture medium (#PM150110, Procell, Wuhan, China) supplemented with 10% fetal bovine serum (FBS, Gibco, USA) at 37°C with 5% CO_2_ in a humidified incubator. For some experiments, siRNA‐CCR1 and/or siRNA‐CCR2 were transfected using liposomes LipofectamineTM 2000 (Thermofisher, Waltham, MA, USA). 10 μM AKT inhibitor MK‐2206 was used to treat SNU‐878 cells.

### 2.6 Cell Counting Kit 8 Assay

Cells were seeded in 96‐well plates at an initial density of 5 × 10^3^ cells/well. After 12 h, different concentrations of CCL7 protein (Peprotech, USA, 0, 2.5, 5.0, 12.5, 25.0, 50.0 ng/mL) were added to the medium. The cell viability of SNU‐878 cells was measured using the Cell Counting Kit 8 (CCK‐8, Thermo Fisher Scientific) according to the manufacturer's instructions. Absorbance was recorded at 450 nm.

### 2.7 Clonogenic Assay

SNU‐878 cells (1.0 × 10^3^ cells/well) were seeded in six‐well plates and incubated at 37°C with 5% CO_2_ for 2 weeks. Following incubation with 25.0 ng/mL CCL7 protein, the cells were fixed with formaldehyde and then stained with 0.1% crystal violet for 5 min at room temperature. The numbers of colonies (> 50 cells) were counted using an optical inverted microscope (Olympus Corporation).

### 2.8 Transwell Assay

#### 2.8.1 Migration Experiment

SNU‐878 cells were treated with 25.0 ng/mL CCL7 protein. Then, 1640 medium without FBS was used to adjust the cell concentration to 2 × 10^5^ cells/mL. 200 μL was added into the upper chamber of Transwell, and then 700 μL 1640 medium containing 10% FBS was added into the lower chamber. Incubated at 37°C and 5% CO_2_ for 24 h, the cells in the upper chamber were carefully removed with a cotton swab, and the migrated cells were fixed with methanol for 30 min, stained with 0.1% crystal violet for 20 min, and observed in five fields randomly selected by an inverted microscope (Olympus Corporation).

#### 2.8.2 Invasion Experimen0t

2 × 10^4^ cells were inoculated into the upper chamber of Transwell pre‐coated with Matrigel, and then 1640 culture solution with 10% FBS was added to the lower chamber. The remaining steps migrate with the above cells.

### 2.9 Wound Healing Assay

The migratory capacity of SNU‐878 cells was assessed utilizing the Wound Healing Assay technique. Cells were cultured in a 6‐well plate until they achieved 90% confluence. At this juncture, a sterile micropipette tip was employed to introduce a wound in the cell monolayers. The wounded monolayers were subsequently rinsed with PBS to remove any cellular debris. The distance between the wound edges was measured at three separate locations, with a follow‐up measurement conducted 24 h post‐wounding to evaluate cell migration.

### 2.10 Immunofluorescence (IF) Staining

For IF analysis, the cells were treated with 25.0 ng/mL CCL7 protein for 48 h. Then, the cells were washed twice in PBS for 5 min each. Subsequently, the cells were incubated overnight at 4°C with the primary antibody, namely E‐cadherin (#GB12082, Servicebio, Wuhan, China; 1/100), N‐cadherin (#GB12135‐100, Servicebio, Wuhan, China; 1/200), Vimentin (#ab92547, Abcam, CA, USA; 1/200), and MMP2 (#ab97779, Abcam, CA, USA; 1/200). Then, the goat‐ anti‐mouse Cy3 labeled (#GB21301; Servicebio, Wuhan, China; 1:100) and FITC‐conjugated goat anti‐rabbit (#GB22303; Servicebio, Wuhan, China; 1:100) secondary antibodies were used. Ultimately, the cells were stained using 4′,6‐diamidino‐2‐phenylindole (DAPI; #G1012; Servicebio, Wuhan, China). The images were visualized and photographed by confocal fluorescence microscopy (Carl Zeiss, Thornwood, NY, USA).

### 2.11 Western Blot Analysis

Liver tissue and SNU‐878 cell lysate solution was fabricated using RIPA buffer (Signaling Technology Inc.). The concentration of protein was determined by a BCA kit (Sigma‐Aldrich; Merck KGaA). Total protein (30 μg/sample) was separated via 10% SDS‐PAGE. Then the separated proteins were transferred to nitrocellulose membranes. The membranes were blocked with 5% nonfat dried milk overnight at 4°C and incubated with the following corresponding protein antibodies: AKT (#10176‐2‐AP, Proteintech, Wuhan, China; 1/2000), phosphor(p)‐AKT (#66444‐1‐Ig, Proteintech, Wuhan, China; 1/5000), PI3K (#67071‐1‐Ig, Proteintech, Wuhan, China; 1/2000), p‐PI3K (#AF3241, Proteintech, Wuhan, China; 1/2000), and β‐actin (#AC026, ABclonal, Wuhan, China; 1/50000). Then, the membranes were washed with Tris‐buffered saline/0.1% Tween (TBST) and incubated for 1.5 h with an HRP Goat anti‐Rabbit IgG (Abcam, ab6721). The bands were visualized using the ECL system (Affinity Biosciences, Cincinnati, Ohio, USA) and β‐actin was used as an internal control. The net optical density was measured using Quantity One software (Bio‐Rad, CA, USA).

### 2.12 Enzyme‐Linked Immunosorbent Assay (ELISA)

SNU‐878 cells were exposed to CCL7 for ELISA. The contents of VEGF in culture supernatants of SNU‐878 cells were measured with ELISA kits (#ZC‐35248, ZCi Bio, Shanghai, China), following the manufacturer's instructions. The absorbance was measured at a 450 nm wavelength and was estimated using an enzyme‐linked immune monitor (Thermo Fisher Scientific Inc., USA). The concentration of VEGF in the sample was calculated from the standard curve.

### 2.13 Statistical Analysis

The data were represented as means ± standard deviation. Statistical analysis was performed using SPSS 20.0 (IBM Corp.). One‐way analysis of variance (ANOVA) with Tukey's post hoc test of means was used for comparison between groups. Differences with a *p* < 0.05 were considered to indicate statistical significance.

## 3 Results

### 3.1 The Expression of CCL7 Was Up‐Regulated in HCC Tissue and Portal Vein Cancer Embolus Tissue

**FIGURE 1:**
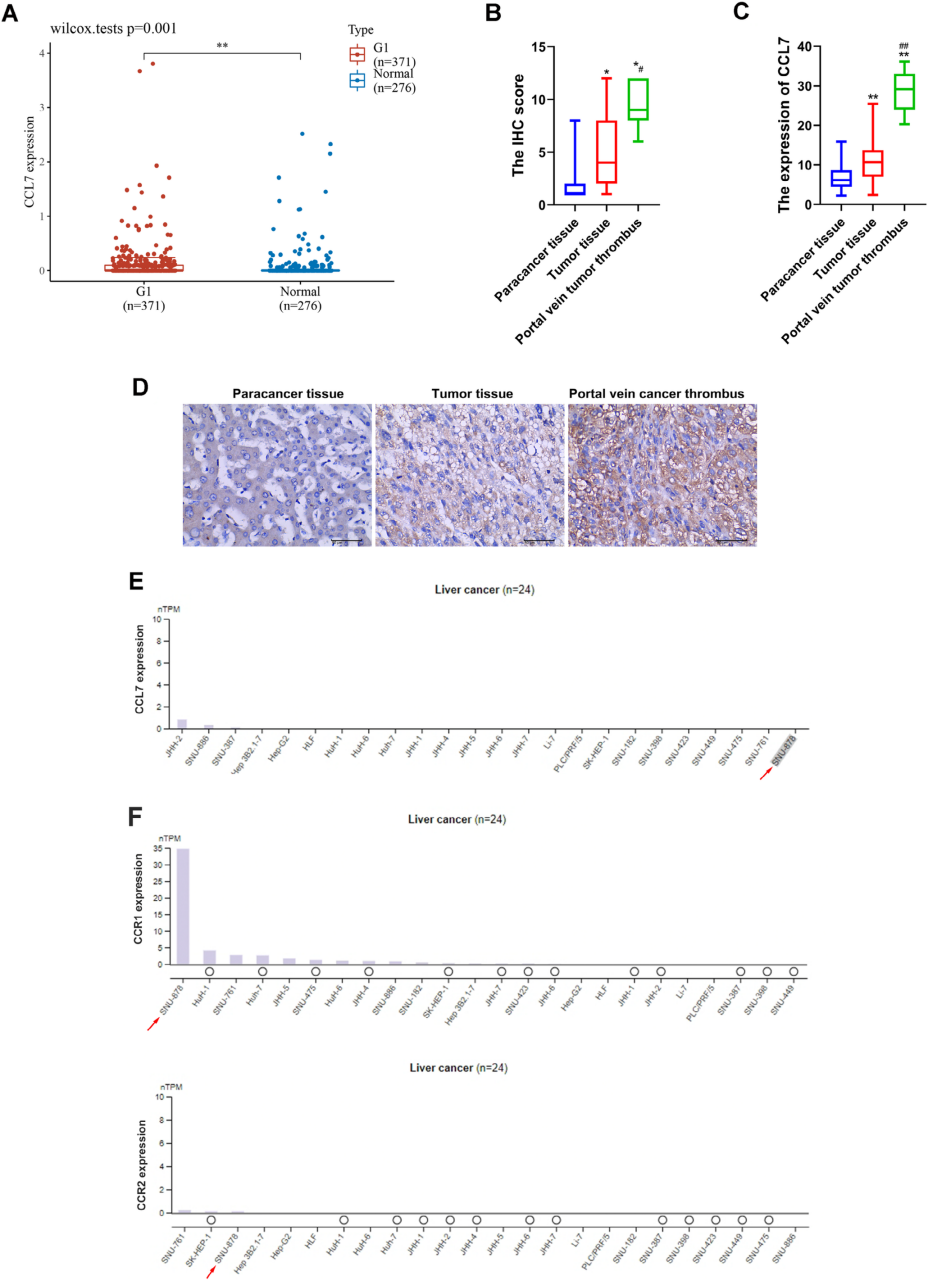
The expression of CCL7 was up‐regulated in HCC tissue and portal vein cancer embolus tissue. (A) Analysis of the expression level of CCL7 in HCC patients using TCGA databases. **p < 0.01 versus G1. (B) The immunohistochemical score was calculated by combining the proportion score (percentage of positive stained cells) with the staining intensity score. (C and D) Immunohistochemical methods were used to detect CCL7 expression in 50 HCC tissues, 49 paracancer tissues, and 10 metastatic carcinomas (portal vein tumor thrombus). (E and F) CCL7, CCR1, and CCR2 mRNA expression in a hepatoma cell line from the Human Protein Atlas (https://www.proteinatlas.org). *p < 0.05, **p < 0.01 versus paracancer tissue. ^#^ *p* < 0.05, ^##^ *p* < 0.01 versus tumor tissue.

TCGA database from HOME for Researchers platform (https://www.home‐for‐researchers.com/) showed that the level of CCL7 was significantly up‐regulated in HCC tissue compared with normal liver tissue (Figure 1A). There were significant differences in IHC scores between the tumor tissue and adjacent tissue (Figure 1B). Furthermore, IHC staining showed that the expression of CCL7 was increased in HCC tumor tissue and portal vein tumor thrombus tissue compared with paracancer tissue (Figure 1C,D). Also, compared with carcinoma in situ, the expression of CCL7 in portal vein cancer embolus tissue was further increased (Figure 1C,D). We also acquired the CCL7 mRNA expression in liver cancer from the Human Protein Atlas (https://www.proteinatlas.org). However, hepatoma cell lines did not express CCL7 (Figure 1E). In addition, we acquired the CCL7 mRNA expression in liver cancer from the Human Protein Atlas (https://www.proteinatlas.org). However, hepatoma cell lines did not express CCL7 (Figure 1E). Additionally, we investigated the mRNA expression of CCR1 and CCR2 in HCC using data from the Human Protein Atlas (Figure 1F). Notably, CCR1 exhibited high expression levels in the HCC cell line SNU‐878, whereas CCR2 was not detected in this cell line (Figure 1F).

### 3.2 The Up‐Regulated Expression of CCL7 in HCC Was Associated With the Adverse Pathological Features of HCC

Based on the median expression index of 4.0, determined by the IHC score for CCL7 protein expression levels in a cohort of 50 patients with hepatocellular carcinoma (HCC), the patients were categorized into two groups: CCL7‐strongly positive and CCL7‐weakly positive. The results of the chi‐square test analysis indicated that, in comparison to the CCL7‐weakly positive group, the CCL7‐strongly positive group exhibited tumors that were larger in size, less differentiated, more frequently associated with regional lymph node metastasis or distant metastasis, and characterized by a more advanced TNM stage (Table 1).

**TABLE 1:** Association between CCL7 and clinicopathological characteristics of HCC patients.

Variables	Cases (*n* = 50)	CCL7 high (*n* = 29)	CCL7 low (*n* = 21)	*p*
Age (years)				
< 49	15	11 (37.9%)	4 (19%)	0.215
≥ 49	35	18 (62.1%)	17 (81%)	
Sex				
Male	42	23 (79.3%)	19 (90.5%)	0.441
Female	8	6 (20.7%)	2 (9.5%)	
HBV				
Negative	9	7 (24.1%)	2 (9.5%)	0.271
Positive	41	22 (75.9%)	19 (90.5%)	
AFP (ng/mL)				
< 20	13	6 (20.7%)	7 (33.3%)	0.314
≥ 20	37	23 (79.3%)	14 (66.7%)	
Cirrhosis				
Yes	21	13 (44.8%)	8 (38.1%)	0.634
No	29	16 (55.2%)	13 (61.9%)	
Tumor size				
< 5 cm	18	7 (24.1%)	11 (52.4%)	0.040*
≥ 5 cm	32	22 (75.9%)	10 (47.6%)	
Tumor growth characteristics				
Single	30	16 (55.2%)	14 (66.7%)	0.413
Multiple	20	13 (44.8%)	7 (33.3%)	
Tumor differentiation				
Poorly−/un‐differentiated	21	16 (55.2%)	5 (23.8%)	0.027*
Well/moderately differentiated	29	13 (44.8%)	16 (76.2%)	
Microvascular invasion				
No	29	14 (48.3%)	15 (71.4%)	0.102
Yes	21	15 (51.7%)	6 (28.6%)	
Lymph node or distant metastasis				
No	43	21 (100%)	22 (75.9%)	0.017*
Yes	7	0	7 (24.1%)	
TNM stage				
I–II	22	9 (31%)	13 (61.9%)	0.030*
III–IV	28	20 (69%)	8 (38.1%)	

* *p* < 0.05.

### 3.3 Exogenous CCL7 Promoted the Growth of HCC Cells and the Activation of the PI3K/AKT Pathway

**FIGURE 2:**
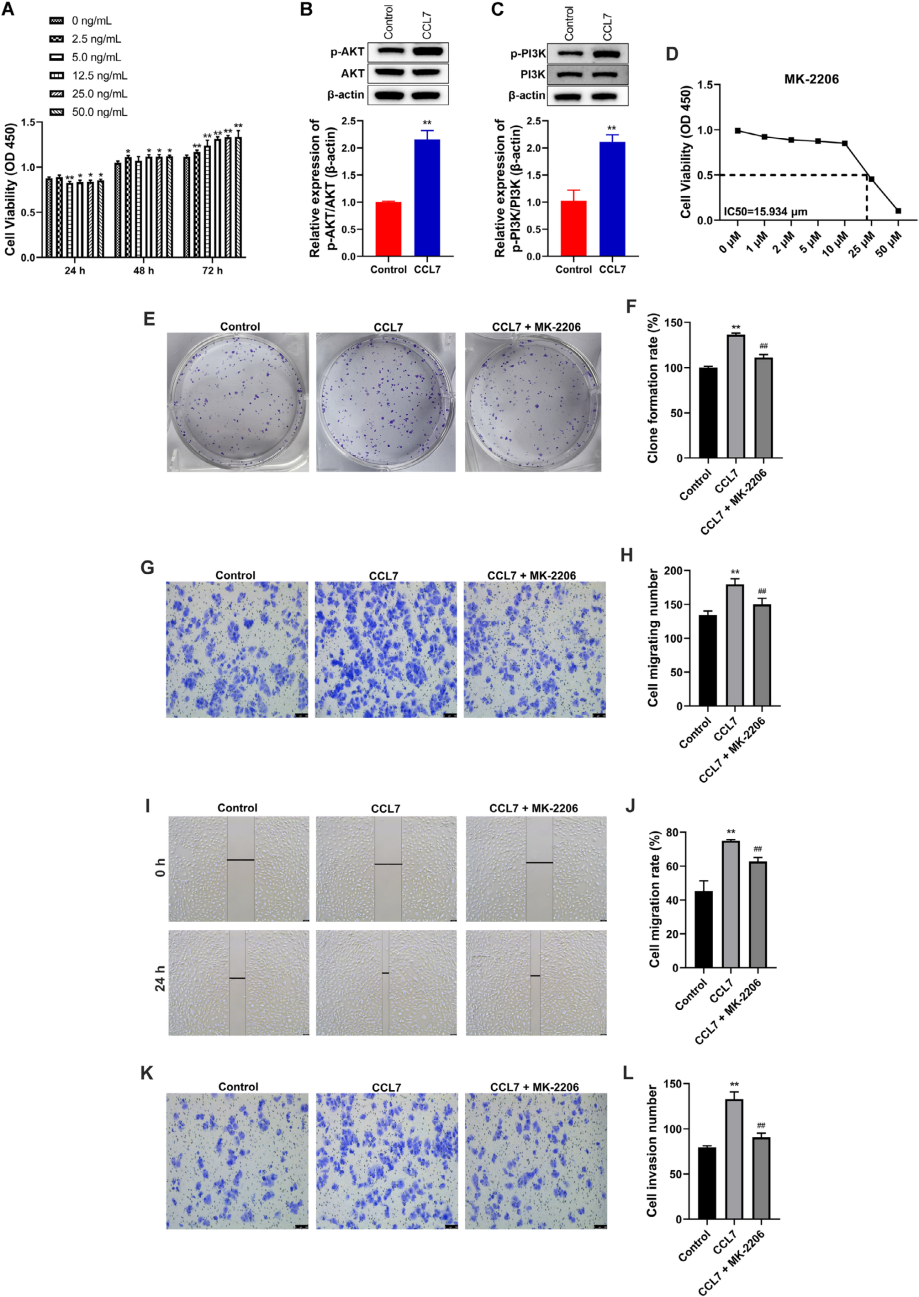
Exogenous CCL7 promoted the proliferation, migration, and invasion of HCC cells. SNU‐878 cells were treated by CCL7 or/and AKT inhibitor MK‐2206. (A) The growth of SNU‐878 cells was detected by CCK‐8. *p < 0.05, **p < 0.01 versus 0 ng/mL. (B and C) Western blot was used to assay determined PI3K, p‐PI3K, AKT, and p‐AKT expression. (D) SNU‐878 cells were treated with increasing concentrations of MK‐2206. Cell proliferation was measured using CCK‐8. (E and F) The effect of CCL7 on the proliferation of SNU‐878 cells was detected by clonogenic assay. (G and H) The effect of CCL7 on SNU‐878 cell migration was detected by transwell assay. (I and J) The effect of CCL7 on SNU‐878 cell migration was detected by wound healing assay. (K and L) The effect of CCL7 on SNU‐878 cell invasion was observed by Transwell assay. **p < 0.01 versus Control. ^##^ *p* < 0.01 versus CCL7.

**FIGURE 3:**
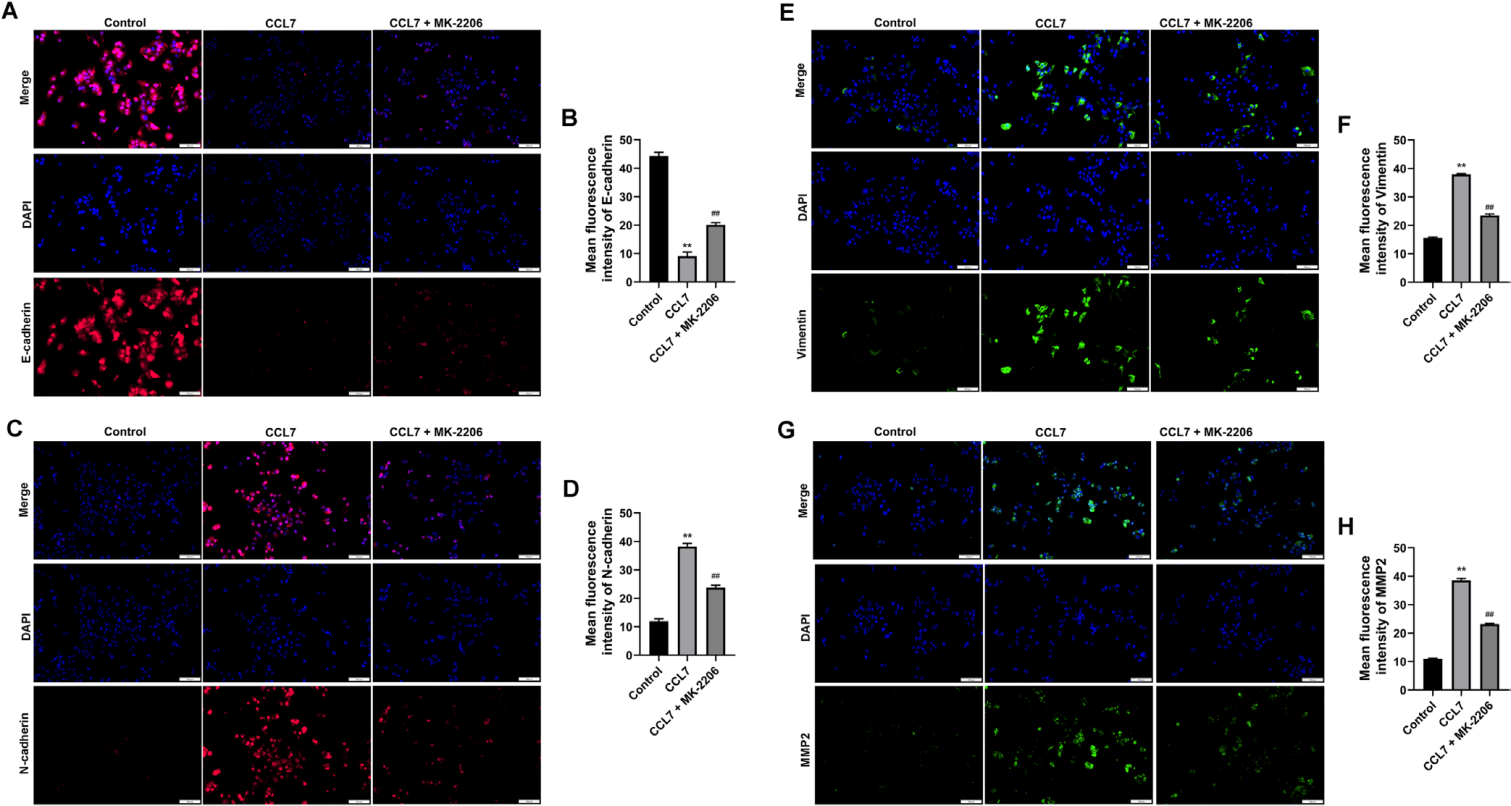
Exogenous CCL7 increased the expression of EMT‐associated proteins in HCC cells. SNU‐878 cells were treated by CCL7 or/and AKT inhibitor MK‐2206. (A–H) Expression levels of E‐cadherin, N‐cadherin, vimentin, and MMP9 in SNU‐878 cells were detected using an Immunofluorescence staining (magnification 100×). **p < 0.01 versus Control. ^##^ *p* < 0.01 versus CCL7.

As illustrated in Figure 2A, the addition of exogenous CCL7 significantly enhanced the proliferation of SNU‐878 cells in a manner dependent on both concentration and time. Notably, the most pronounced proliferation was observed in cells treated with 25 ng/mL CCL7 at both 48 and 72 h. Consequently, subsequent experiments utilized a concentration of 25 ng/mL CCL7 to further assess its impact on hepatocyte function. Furthermore, CCL7 was found to promote the phosphorylation of PI3K and AKT proteins in SNU‐878 cells, as depicted in Figure 2B,C. Next, the AKT inhibitor MK‐2206 was co‐treated with CCL7 to evaluate the effect of CCL7 on the growth of SNU‐878 cells through the PI3K/AKT signaling pathway. As shown in Figure 2D, the IC50 of MK‐2206 on SNU‐878 cells was ascertained by exposing the cells to MK‐2206 for a duration of 24 h. Subsequently, cell proliferation was assessed using the CCK‐8 assay, resulting in the determination of the IC50 value as 15.934 μM. The clonogenic assay suggested that CCL7 induction significantly promoted the proliferation of SNU‐878 cells, which was reversed by 10 μM MK‐2206 treatment (Figure 2E,F). Transwell and wound healing assays showed that CCL7‐induced increased cell migration and invasion were also hindered by MK‐2206 treatment (Figure 2G–L). Epithelial‐mesenchymal transition (EMT)‐related proteins in HCC cells were evaluated by IF staining. The data suggested that CCL7 reduced the expression of E‐cadherin (Figure 3A,B), and increased the protein levels of N‐cadherin (Figure 3C,D), vimentin (Figure 3E,F) and MMP2 (Figure 3G,H). Compared with the CCL7‐treated group, MK‐2206 further induced the expression of E‐cadherin (Figure 3A,B) and decreased the protein levels of N‐cadherin (Figure 3C,D), vimentin (Figure 3E,F) and MMP2(Figure 3G,H).

**FIGURE 4:**
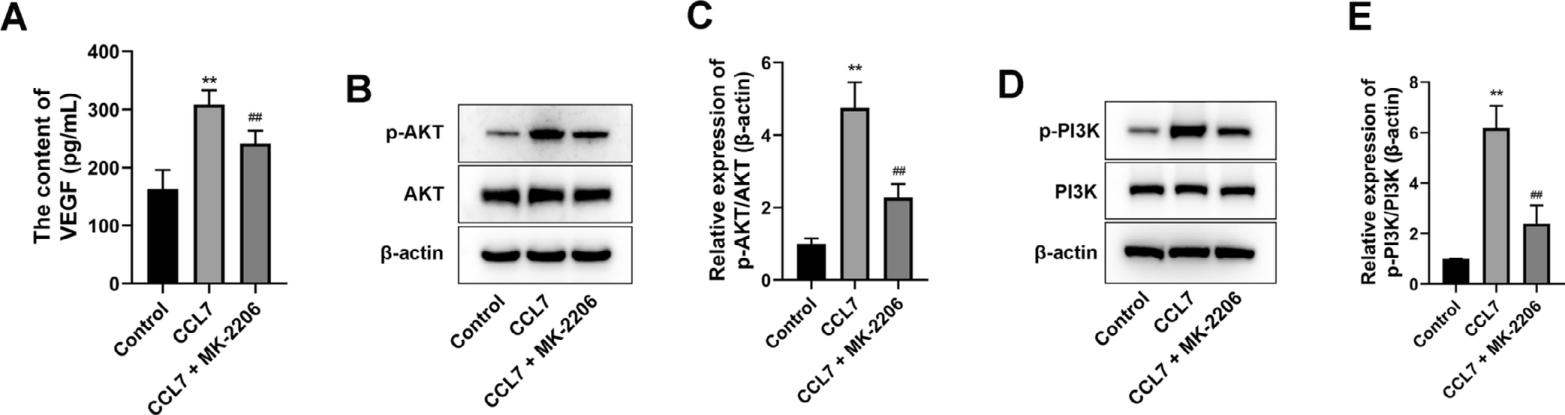
Exogenous CCL7 enhanced the VEGF secretion and PI3K/AKT signaling pathway activation in HCC cells. SNU‐878 cells were treated by CCL7 or/and AKT inhibitor MK‐2206. (A) VEGF secretion by SNU‐878 cells was detected by ELISA assay. (B–E) Western blot was performed to detect AKT, p‐AKT, PI3K, and p‐PI3K expression. **p < 0.01 versus control. ^##^ *p* < 0.01 versus CCL7.

The secretion of VEGF (responsible for angiogenesis) by SNU‐878 cells was significantly increased by CCL7 treatment, which was blocked by MK‐2206 (Figure 4A). Furthermore, the proteins of the PI3K/AKT signaling pathway, namely PI3K, p‐PI3K, AKT, and p‐AKT, were subjected to analysis using a Western blot technique. It was found that after CCL7 treatment alone, the expressions of p‐PI3K and p‐AKT were markedly promoted, and the ratios of p‐PI3K/PI3K and p‐AKT/AKT were up‐regulated in SNU‐878 cells (Figure 4B–E). In addition, compared with the CCL7‐treated group, MK‐2206 further inhibited the expression of p‐PI3K and p‐AKT, and the ratios of p‐PI3K/PI3K and p‐AKT/AKT in SNU‐878 cells (Figure 4B–E).

### 3.4 CCL7 Promoted the Growth of HCC Cells and the Activation of the PI3K/AKT Pathway by Targeting CCR1 and CCR2

**FIGURE 5:**
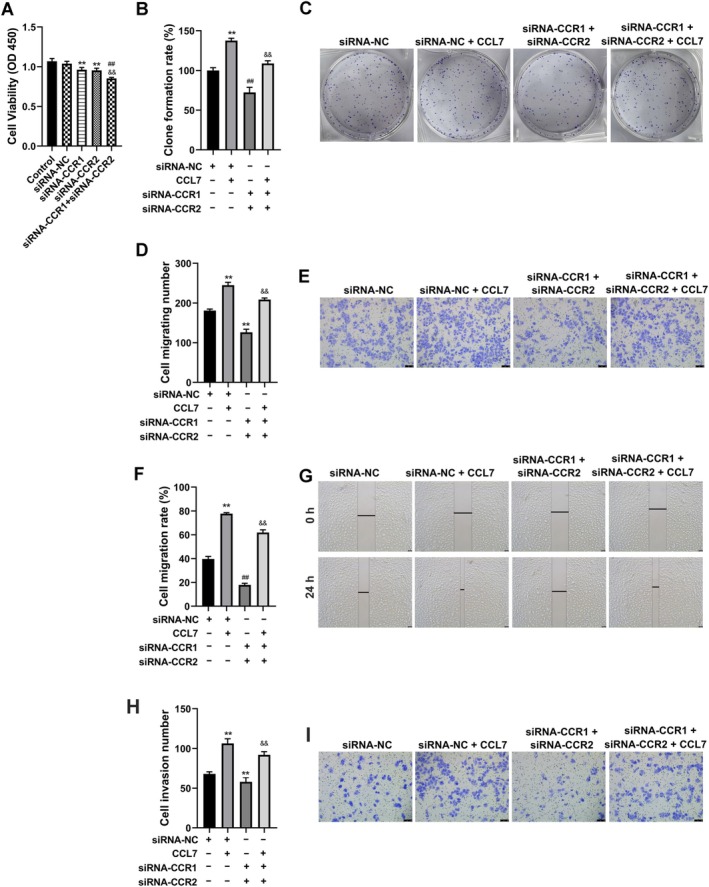
CCL7 promoted the growth of HCC cells by targeting CCR1 and CCR2. SNU‐878 cells were treated with CCL7, while SNU‐878 cells were transfected with siRNA‐CCR1 and siRNA‐CCR2. (A) The growth of SNU‐878 cells was detected by CCK‐8. **p < 0.01 versus siRNA‐NC. ^##^ *p* < 0.01 versus siRNA‐CCR1. ^&&^ *p* < 0.01 versus siRNA‐CCR2. (B and C) The proliferation of SNU‐878 cells was detected by clonogenic assay. (D and E) SNU‐878 cell migration was detected by Transwell assay. (F and G) SNU‐878 cell migration was detected by wound healing assay. (H and I) SNU‐878 cell invasion was observed by the Transwell assay. **p < 0.01 versus siRNA‐NC. ^##^ *p* < 0.01 versus CCL7.

CCR1 and CCR2 have been identified as potential receptors for CCL7. Consequently, we explored the potential role of CCL7 in modulating the growth of SNU‐878 cells via CCR1 and CCR2. As illustrated in Figure 5A, the knockdown of CCR1 and/or CCR2 in SNU‐878 cells resulted in a marked reduction in cell proliferation. Furthermore, the clonogenic assay demonstrated that the CCL7‐induced proliferation of cells was significantly attenuated by the simultaneous application of siRNA targeting CCR1 and CCR2 (Figure 5B,C). Transwell and wound healing assays suggested that CCL7 promoted the migration and invasion of SNU‐878 cells, while siRNA‐CCR1+ siRNA‐CCR2 inhibited the migration and invasion of SNU‐878 cells (Figure 5D–I). At the same time, the increase in cell migration and invasion induced by CCL‐7 was significantly hindered by siRNA‐CCR1+ siRNA‐CCR2 co‐treatment (Figure 5D–I).

**FIGURE 6:**
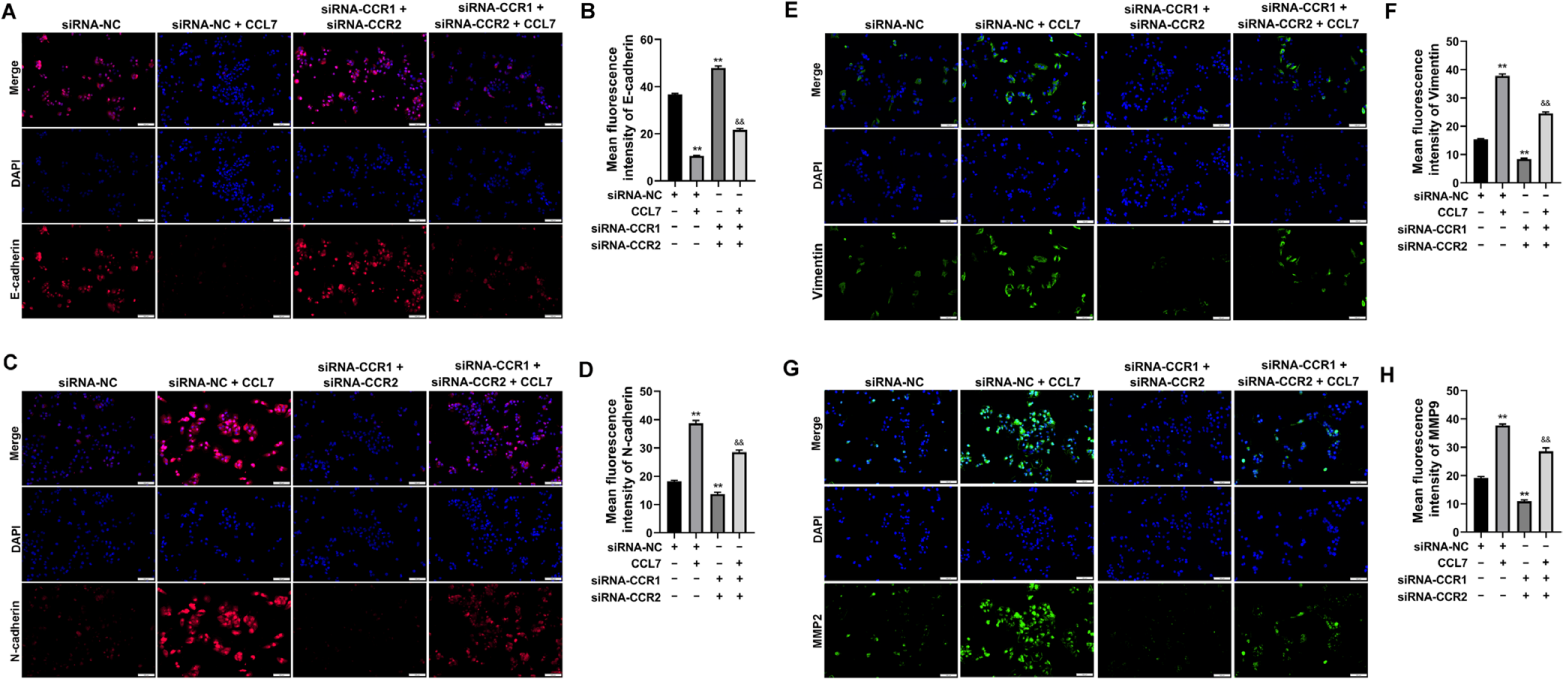
CCL7 increased the expression of EMT‐associated proteins in HCC cells by targeting CCR1 and CCR2. SNU‐878 cells were treated with CCL7, while SNU‐878 cells were transfected with siRNA‐CCR1 and siRNA‐CCR2. (A–H) Expression levels of E‐cadherin, N‐cadherin, vimentin, and MMP9 in SNU‐878 cells were detected using an Immunofluorescence staining (magnification 100×). **p < 0.01 versus siRNA‐NC. ^&&^ *p* < 0.01 versus siRNA‐NC +CCL7.

IF staining suggested that CCL7 resulted in reduced E‐cadherin (Figure 6A,B) and increased N‐cadherin (Figure 6C,D), vimentin (Figure 6E,F) and MMP2 (Figure 6G,H) expression, while siRNA‐CCR1 + siRNA‐CCR2 silencing increased E‐cadherin (Figure BA and 6B) and reduced N‐cadherin (Figure 6C,D), vimentin (Figure 6E,F) and MMP2 expression in SNU‐878 cells (Figure 6G,H). Compared with the, siRNA‐CCR1 + siRNA‐CCR2 + CCL7 co‐treatment further induced the expression of E‐cadherin (Figure 6A,B), and decreased the protein levels of N‐cadherin (Figure 6C,D), vimentin (Figure 6E,F) and MMP2 in SNU‐878 cells (Figure 6G,H).

**FIGURE 7:**
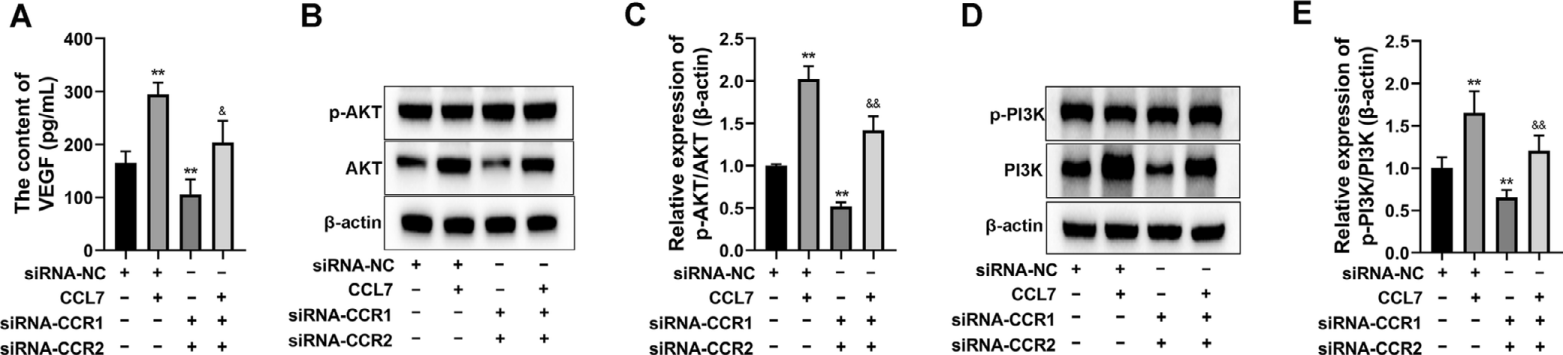
CCL7 enhanced the VEGF secretion and PI3K/AKT signaling pathway activation in HCC cells by targeting CCR1 and CCR2. SNU‐878 cells were treated with CCL7, while SNU‐878 cells were transfected with siRNA‐CCR1 and siRNA‐CCR2. (A) VEGF secretion by SNU‐878 cells was detected by ELISA assay. (B–E) Western blot was performed to detect AKT, p‐AKT, PI3K, and p‐PI3K expression. **p < 0.01 versus siRNA‐NC. ^&^ *p* < 0.05, ^&&^ *p* < 0.01 versus siRNA‐NC + CCL7.

Then, the secretion of VEGF by SNU‐878 cells was significantly induced by CCL7 treatment, which was blocked by siRNA‐CCR1+ siRNA‐CCR2 co‐treatment (Figure 7A). The protein expression levels of PI3K, p‐PI3K, AKT, and p‐AKT in the PI3K/AKT signaling pathway were detected by Western Blot (Figure 7B–E). We found that the protein expression levels of p‐PI3K and p‐AKT were increased in the CCL7‐treated group and were decreased in the siRNA‐CCR1 + siRNA‐CCR2‐transfected group in SNU‐878 cells (Figure 7B–E). Moreover, the increased p‐PI3K and p‐AKT expressions induced by CCL7 were significantly reversed by siRNA‐CCR1+ siRNA‐CCR2 co‐treatment (Figure 7B–E).

### 3.5 CCL7 Facilitated HCC Tumorigenesis In Vivo

**FIGURE 8:**
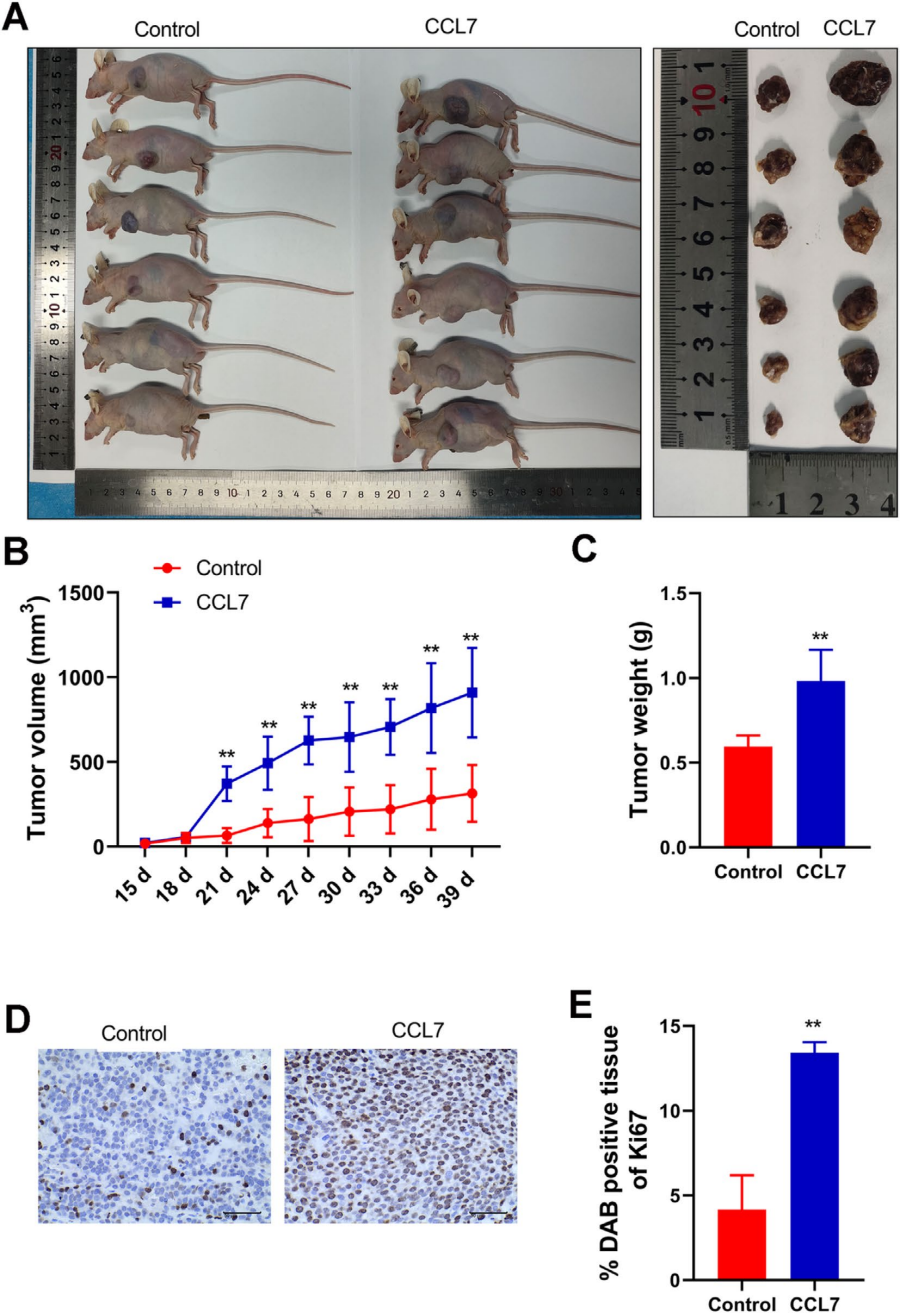
CCL7 facilitated HCC tumorigenesis in vivo. (A–C) Compared with the control group, CCL7 promoted the tumor volume and tumor weight. (D and E) IHC staining of Ki67, magnification is 400×. **p < 0.01, versus control.

**FIGURE 9:**
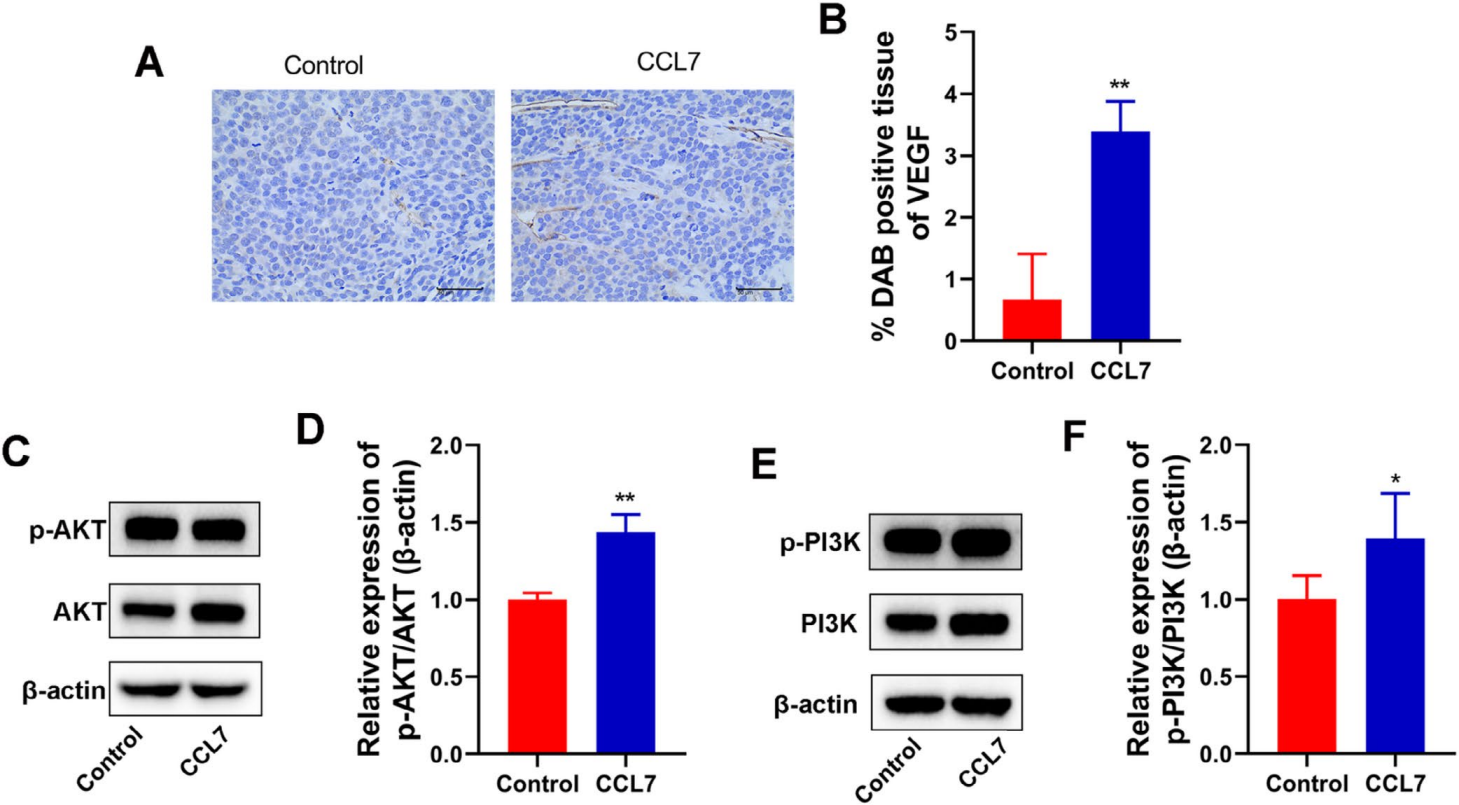
CCL7 facilitated PI3K/AKT signaling pathway activation in vivo. (A and B) (H) IHC staining of VEGF, magnification is 400×. (C–F) Western blot was used to assay determined AKT, p‐AKT, PI3K, and p‐PI3K expression. *p < 0.05, **p < 0.05, versus control.

To examine the effect of CCL7 in vivo, we subcutaneously injected SNU‐878 cells to generate a xenograft nude mouse model. The treatment of CCL7 increased tumor volume and tumor weight in nude mice (Figure 8A–C). Meanwhile, the expression of a proliferation marker protein Ki67 was increased by CCL7 (Figure 8D,E). Our study further confirmed that CCL7 facilitated tumor growth in vivo. IHC staining of the tumor tissues demonstrated that the positive rate of VEGF in the tumor tissues was greatly increased in the CCL7‐treated group compared with the control group (Figure 9A,B). In addition, Western Blot analysis showed that CCL7 increased the expressions of p‐PI3K and p‐AKT in tumor tissues compared with the control group.

## 4 Discussion

In this study, the objective was to investigate the impact of CCL7 on HCC cell metastasis through the utilization of both in vitro and in vivo methodologies. Initially, it was discovered that CCL7 facilitated the proliferation of HCC cells and stimulated both cell invasion and migration in vitro. Subsequently, in ectopic mouse models, it was observed that cells induced by CCL7 exhibited significantly accelerated growth compared to control cells. Additionally, the induction of cell migration and invasion by CCL7 demonstrated a correlation with CCR1 and CCR2. Lastly, the activation of the PI3K/AKT signaling pathway was observed upon CCL7 stimulation.

Tumor evolution is governed by two primary factors: the intrinsic properties of the tumor itself and the interactions between tumor cells and stromal cells within the surrounding tumor microenvironment. Tumor metastasis is the predominant cause of mortality in patients with liver cancer [20]. EMT is a key step in the early stages of distant metastasis of HCC, during which epithelial cells lose their polarity, acquire enhanced invasive and migratory capabilities, and degrade the extracellular matrix [21, 22]. Cancer cells disseminate to distant regions via the bloodstream, lymphatic fluid, bile duct system, or through direct infiltration into peripheral organs [23]. On the other hand, tumor cells and stromal cells can release a diverse array of cytokines, encompassing growth factors, chemokines, and cytokines, among others. These secreted factors possess the ability to engage with specific signaling pathways either through their receptors or receptors present on neighboring cells, consequently exerting an influence on cellular proliferation, migration, transformation, and other biological processes. Chemokines, such as CCL7, CCL2, CCL5, etc., belong to a group of proteins capable of facilitating the targeted movement of immune cells. Through their interaction with specific receptors, such as CCR1, CCR2, CCR3, etc., these chemokines play a crucial role in the evolution of liver cancer [24, 25]. The inhibition of CCL2/CCR2 signaling effectively hinders the growth of liver tumors in mice by stimulating the antitumor immune response of T cells [26]. The promotion of hepatocellular carcinoma metastasis was also facilitated by the activation of the HIF1α/ZEB1 axis through CCL5 derived from cancer‐associated fibroblasts [27]. Research indicates that cancer‐associated fibroblasts (CAFs) secrete elevated levels of CCL2, CCL5, CCL7, and CXCL16, which facilitate the migration and invasion of HCC cells [28]. Furthermore, CCL2 and CCL5 have been shown to activate the hedgehog (Hh) signaling pathway, whereas CCL7 and CXCL16 enhance the activity of the transforming growth factor‐β (TGF‐β) pathway in HCC cells [28]. Notably, our findings demonstrate that CCL7 expression is significantly higher in HCC tumor tissues compared to adjacent non‐tumorous tissues. However, liver cancer cells exhibit a limited capacity to secrete CCL7. Therefore, our experiment proved that exogenous CCL7 promoted the migration, invasion, and EMT of HCC cells by binding to the CCR1/2 receptor. Meanwhile, for the first time, we found that CCL7 induced the growth of HCC tumors in vivo.

In addition, our study also showed that CCL7 promoted VEGF by activating the CCR1/2 receptor. VEGF, a pivotal molecule within the tumor microenvironment, is synthesized by tumor cells and various other cell types to facilitate angiogenesis, thereby significantly contributing to tumor progression and metastasis [29, 30]. Within the tumor microenvironment, VEGF secretion effectively stimulates the proliferation and migration of vascular endothelial cells, thereby fostering neovascularization and facilitating the provision of essential nutrients and oxygen to the tumor [31]. Furthermore, VEGF exerts influence over immune cell activation and functionality, effectively suppressing immune responses and aiding tumor cells in evading immune surveillance [32]. Previous reports demonstrated that activation of the PI3K/AKT signaling pathway was closely associated with vascular remodeling and angiogenesis in HCC. According to the report, apatinib has been demonstrated to reduce the expression of VEGF and PI3K/AKT, thereby exerting inhibitory effects on angiogenesis and the invasion of HCC cells [33]. Furthermore, asparagus polysaccharide (ASP) was shown to down‐regulate the phosphorylation levels of PI3K and AKT, resulting in the suppression of the HIF‐1α/VEGF axis, thereby inhibiting angiogenesis and hindering the progression of HCC cells [34]. Consequently, this study explores the regulatory role of CCL7 on the PI3K/AKT signaling pathway. We showed that CCL7 induced the secretion of VEGF by promoting the PI3K/AKT signaling pathway. In addition to angiogenesis, PI3K/AKT signaling increased clonogenic ability and promoted tumor cell spreading [35]. During HCC metastasis, PI3K/AKT stimulates EMT and increases MMP expression [36]. Therefore, silencing PI3K/AKT signaling prevented aggressive HCC cell behavior. Similarly, the present study also found that silencing PI3K/AKT signaling reversed the promotional effects of CCL7 on HCC cell proliferation, migration, and invasion.

Our findings collectively indicate that the activation of the PI3K/AKT signaling pathway, which is associated with CCR1/2, may constitute a molecular mechanism by which CCL7 facilitates growth and metastasis in HCC. The evidence of this mechanistic interaction between the CCL7/CCR1/2 axis and the PI3K/AKT cascade offers significant insights into various cellular processes such as proliferation, invasion, migration, and angiogenesis in HCC. Consequently, these findings propose that CCL7 could be a viable therapeutic target for the treatment of HCC. Further studies are needed to validate these findings and explore the potential therapeutic applications of CCL7 inhibition in HCC.

## Author Contributions

**Yangkun Luo:** conceptualization (lead), data curation (lead), formal analysis (lead), investigation (lead), methodology (lead), software (equal), validation (lead), writing – original draft (lead). **Fei Wan:** data curation (equal), formal analysis (equal), investigation (equal), software (equal), validation (equal). **Zhao Zhang:** data curation (equal), formal analysis (equal), software (equal). **Zujun Qin:** data curation (equal), formal analysis (equal), investigation (equal), validation (equal). **Yongjun Ren:** conceptualization (equal), funding acquisition (lead), methodology (equal), project administration (lead), resources (lead), supervision (lead), writing – review and editing (equal).

## Conflicts of Interest

The authors declare no conflicts of interest.

## Data Availability

The datasets used or analyzed during the current study are available from the corresponding author upon reasonable request.
